# Radiomics analysis of T1WI and T2WI magnetic resonance images to differentiate between IgG4-related ophthalmic disease and orbital MALT lymphoma

**DOI:** 10.1186/s12886-023-03036-7

**Published:** 2023-06-23

**Authors:** Yuchao Shao, Yuqing Chen, Sainan Chen, Ruili Wei

**Affiliations:** Department of Ophthalmology, Shanghai Changzheng Hospital, Shanghai, China

**Keywords:** Machine learning, IgG4-ROD, Orbital MALT lymphoma, SVM

## Abstract

**Background:**

Preoperative differentiation between IgG4-related orbital disease (IgG4-ROD) and orbital mucosa-associated lymphoid tissue (MALT) lymphoma has a significant impact on clinical decision-making. Our research aims to construct and evaluate a magnetic resonance imaging (MRI)-based radiomics model to assist clinicians to better identify IgG4-ROD and orbital MALT lymphoma and make better preoperative medical decisions.

**Methods:**

MR images and clinical data from 20 IgG4-ROD patients and 30 orbital MALT lymphoma patients were classified into a training (21 MALT; 14 IgG4-ROD) or validation set (nine MALT; six IgG4-ROD). Radiomics features were collected from T1-weighted (T1WI) and T2-weighted images (T2WI). Student’s t-test, the least absolute shrinkage and selection operator (LASSO) and principal component analysis (PCA) were conducted to screen and select the radiomics features. Support vector machine (SVM) classifiers developed from the selected radiomic features for T1WI, T2WI and combined T1WI and T2WI were trained and tested on the training and validation set via five-fold cross-validation, respectively. Diagnostic performance of the classifiers were evaluated with area under the curve (AUC) readings of the receiver operating characteristic (ROC) curve, and readouts for precision, accuracy, recall and F1 score.

**Results:**

Among 12 statistically significant features from T1WI, four were selected for SVM modelling after LASSO analysis. For T2WI, eight of 51 statistically significant features were analyzed by LASSO followed by PCA, with five features finally used for SVM. Combined analysis of T1WI and T2WI features selected two and four, respectively, for SVM. The AUC values for T1WI and T2WI classifiers separately were 0.722 ± 0.037 and 0.744 ± 0.027, respectively, while combined analysis of T1WI and T2WI classifiers further enhanced the classification performances with AUC values ranging from 0.727 to 0.821.

**Conclusion:**

The radiomics model based on features from both T1WI and T2WI images is effective and promising for the differential diagnosis of IgG4-ROD and MALT lymphoma. More detailed radiomics features and advanced techniques should be considered to further explore the differences between these diseases.

## Background

Lymphoproliferative disorders are one of the common etiologies that can occur in the orbital space. In an epidemiological survey in Japan, mucosa-associated lymphoid tissue extranodal marginal zone B-cell lymphoma (MALT lymphoma) was the most common subtype of lymphoproliferative diseases, and IgG4-related ophthalmic disease (IgG4-ROD) ranked second [1].

Orbital MALT lymphoma is a low-grade, non-Hodgkin lymphoma subtype, with a low incidence that accounts for only 1% of all non-Hodgkin lymphomas. However, it is one of the most common adult primary orbital tumors, accounting for 55% of orbital malignancies [2]. Common clinical manifestations of MALT lymphoma include periorbital edema, proptosis, strabismus, and diplopia caused by restricted eye movement and other nonspecific manifestations. IgG4-related ophthalmopathy is the manifestation of IgG4-related diseases in the eye, with clinical features of progressively enlarged mass around the lacrimal gland, swelling of the eyelid, with or without proptosis. It is an idiopathic chronic inflammatory change with unclear etiology. The main findings are related to autoimmunity and possible pathogen infection [3, 4]. Typical pathology of IgG4-ROD includes massive infiltration of IgG4 + plasma cells, phlebitis obliterans, and striated fibrosis [5]. In terms of treatment, active surgical treatment with postoperative radiotherapy and chemotherapy are usually used for MALT lymphoma. For IgG4-ROD, surgical treatment is solely for the purpose of improving facial appearance and visual function, whereas systemic glucocorticoids and monoclonal antibodies such as rituximab is normally the preferred treatment. However, although these treatments are effective in controlling the disease, the recurrence rate is relatively high [6–8]. Therefore, differential diagnosis of these two orbital space-occupying lesions is particularly important for selection of appropriate treatment methods.

Due to the non-specific clinical manifestations of MALT lymphoma and IgG4-ROD, differential diagnosis of these two conditions based on serum IgG4 levels and clinical manifestations alone can be challenging [9, 10]. Several studies have attempted to provide other means of differential diagnostic such as metabolomics and imaging manifestations. For example, the arachidonic acid metabolic pathway was shown to play a critical role in IgG4-associated ophthalmopathy, whereas the tricarboxylic acid cycle is more enriched in MALT lymphoma [11]. With magnetic resonance imaging (MRI), the two lesions appear very similar, both showing isointense or slightly lower signal on T1-weighted images (T1WI) and isointense or slightly higher signal on T2-weighted images (T2WI) [12–15].

However, by maximizing the value of acquired images, improving the quantification of image features to fully interpret the image information, and combining with clinical evaluations, it is possible to provide clinicians with more powerful imaging evidence to identify the two diseases, and to guide the selection of more reasonable and efficient treatment for patients.

With increasing digitalization in medicine, artificial intelligence and machine learning has become crucial in this era of big data. Similar to the process of human learning, machine learning can summarize and filter large amount of data to extract the most representative features [16, 17]. Radiomics is a quantitative analysis of medical imaging data and have been in use for more than a decade. Feature extraction and screening techniques are now quite mature and have been fully applied in the study and differential diagnosis of a variety of diseases with significant results [18, 19].

However, as far as we know, radiomics studies to differentiate IgG4-ROD from orbital MALT lymphoma are rare. Considering previous studies have shown that MRI is an effective method for precise visualization of lesions in these two orbital diseases, we seek to determine whether radiomics analysis can improved the differential diagnosis of IgG4-ROD and orbital MALT lymphoma.

## Materials and methods

### Study design and patient selection

The research is a retrospective cross-sectional study approved by the institutional review board of Shanghai Changzheng Hospital. Written consent from patients were waived.

All patients were diagnosed and treated at the Department of Ophthalmology, Shanghai Changzheng Hospital from 2015 to 2021. No history of other orbital diseases, tumors or previous surgeries were present in the patient cohort. The inclusion criteria for orbital MALT lymphoma was extranodal marginal zone B-cell lymphoma of mucosa-associated lymphoid tissue confirmed by postoperative histopathology and pathologically IgG4 staining less than diagnostic criteria for IgG4-ROD. The inclusion criteria for IgG4-ROD was based on the 2020 Japanese diagnostic criteria for IgG4-related diseases [5]. Exclusion criteria: patients with other orbit-related diseases, such as thyroid-associated ophthalmopathy (TAO); patients with other diseases that can lead to elevated serum IgG4, such as systemic lupus erythematosus, systemic vasculitis, and atopic dermatitis; MR images of poor quality; poor patient cooperation. Clinical and image information from 30 patients with orbital MALT lymphoma and 20 patients with IgG4-ROD were finally selected for analysis.

### MRI and manual annotation

**Fig. 1:**
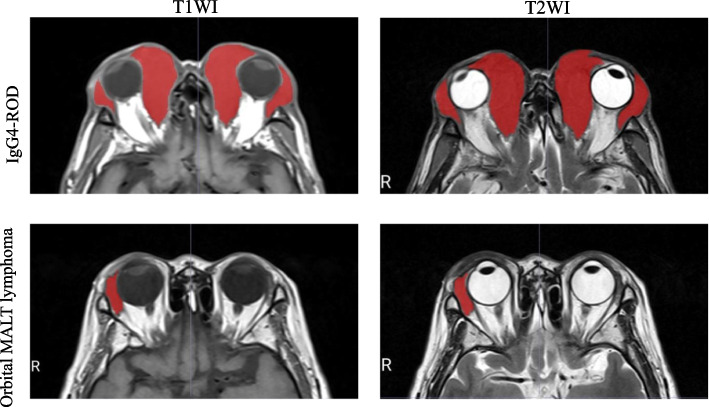
Representative images of annotated ophthalmic lesions. Top row: IgG4-ROD from T1WI (left) and T2WI (right) imaging. Bottom row: orbital MALT lymphoma from T1WI (left) and T2WI (right). Lesion areas are in red

All imaging was performed using a 3.0-T MR equipment (Achieva 3.0T (TX) DS MR system, Philips Healthcare) with imaging parameters shown in Table 1. N4 bias field correction was conducted to avoid inhomogeneity of signal strength and to standardize the images. The ophthalmic lesions were drawn on the images using the MRIcroGL software (version1.2.20211006). These manual annotations were performed by two qualified ophthalmologists with six and 10 years of clinical practice experience in orbital diseases, and whom were blinded to the clinical information and histological diagnosis of the enrolled patients. Figure 1 show representative images of the annotated lesions. Correlation analysis between the annotations performed by these two ophthalmologists was conducted and the interobserver reproducibility of the radiomics features was evaluated by the interclass correlation coefficients (ICC). For images from each MRI sequence, 70% (21 MALT and 14 IgG4-ROD) of the enrolled patients were selected as the training set while the other 30% (9 MALT and 6 IgG4-ROD) formed the validation set.

**Table 1:** Summary of imaging parameters

Parameters	TR (ms)	TE (ms)	TI (ms)	Slice Thickness (mm)	Slices	FA (deg)	Gap (mm)	NSA	b值
Sequence									
T2WI	3000	80		4	16	90	0.4	2	
STIR	4054	80	200	3	16		0.3	2	
T1WI	470	12		3	16	90	0.3	1	
DWI	2500	75		3	16	90	0.3	4	800

### Feature extraction and screening

Using an open-source python package, Pyradiomics (version 3.0.1), 107 radiomics features were extracted from patients’ MR images. These features were selected to represent the lesions and used for radiomics and differential diagnosis by the following analyses. Student’s t-test was first carried out to screen out statistically insignificant imaging features. Then the least absolute shrinkage and selection operator (LASSO) algorithm, which is effective in reducing overfitting and improving the prediction accuracy, was used to identify the optimal features. The regularization parameter (λ) was tuned by the minimum criteria and features were selected using a 10-fold cross-validation method. Radiomics feature weightage maps were drawn using the most important features with non-zero coefficient. In addition, principal component analysis (PCA) was also conducted to further limit the number of radiomics features that would be used to train the predictive model to avoid overfitting caused by redundant features. Finally, four features from T1WI, five from T2WI, and six from combined T1WI and T2WI analyses were selected to construct the predictive models.

### Support vector machine (SVM) training and testing

The radial basis function (RBF) kernel was used for SVM. The parameters were optimized with respect to the training set. The five-fold cross-validation (CV) method (repeated 10 times) was used to train and test the classifiers and the area under the receiver operating characteristic (ROC) curve was used to evaluate their prognostic performance. The entire analytical procedure was accomplished with open-source packages, including Scikit-learn, matplotlib, numpy, pandas and graphviz in Python 3.7.11; PCA was conducted with the R statistical software (version 4.1.3; https://www.r-project.org).

### Statistical analysis

Statistical analyses were performed with the SPSS (version 26.0; SPSS) and R statistical software (version 4.1.3; https://www.r-project.org). The values of precision, accuracy, recall and the F1 score were calculated to evaluate the performance of the diagnosis model. All data with *P* < 0.05 was considered statistically significant.

## Results

### Patient characteristics and tumor distribution

Clinical demographic information of the 50 enrolled patients is shown in Table 2. The difference between these two disease groups is significant. Most orbital MALT lymphomas were unilaterally involved (21/30, 70%), which is consistent with Yuan et al.’s research [20], while IgG4-RODs were mostly bilateral (14/20,70%). However, no significant difference was observed for gender and age between the disease groups. In this study, no conjunctiva involvement was observed in IgG4-RODs while 46.7% MALT lymphomas were conjunctiva involved. Although there was significant difference in the involvement of extraocular muscle and orbital fat between these two diseases, it is still difficult to differentiate since most patients presented 2 or more locations of involvement.

**Table 2:** Patient demographic information

	IgG4 (*n* = 20)	Malt (*n* = 30)	*p* value
Gender			
Male	13	15	
Female	7	15	0.295
Age (years)	57.7 ± 10.3	61.6 ± 7.4	0.078
Ophthalmic involvement			
Unilateral	6	21	
Bilateral	14	9	0.006
Structures involved			
Lacrimal glands	12	10	0.063
Extraocular muscles	7	2	0.029
Orbital fat	8	21	0.035
Conjunctiva	0	14	0.000
Eyelid	6	4	0.279

### Radiomics feature extraction and selection

In total, 107 features were extracted. Among these features, 14 are shape descriptors, including the size and shape of the lesion, which are independent from the gray level intensity distribution in the lesion and are therefore only calculated on the non-derived images and masks. Eighteen features derived from the first-order histogram analysis describe the distribution of the voxel intensities within the image region defined by the mask through commonly used and basic metrics. The remining features all describe different gray level intensity distributions with complex mathematical operations and different algorithms: 24 from gray-level cooccurrence matrix (GLCM) analysis, 16 from gray-level run-length matrix (GLRLM) analysis, 16 from gray-level size zone matrix (GLSZM) analysis, 14 from gray-level differential matrix (GLDM) analysis and five from neighborhood gray tone difference matrix (NGTDM) analysis [21]. The interclass correlation coefficient (ICC) is 0.986 ± 0.001 for IgG4 and 0.983 ± 0.001 for MALT lymphoma, suggesting a high degree of consistency between the two ophthalmologists in annotating these MR images.

**Fig. 2:**
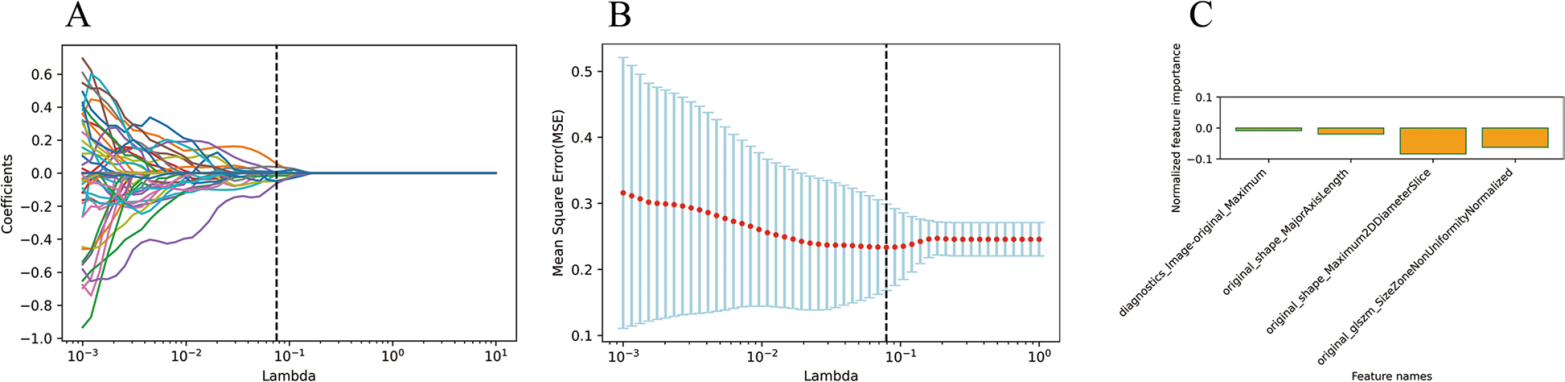
Analyses and selection of radiomics features from T1WI images. **A** LASSO analysis of T1WI. **B** Changes in the automated optimization parameter (λ) when training samples were fitted with LASSO cross-validation (LASSO-CV). **C** The respective weightage of the four selected features for T1WI

**Fig. 3:**
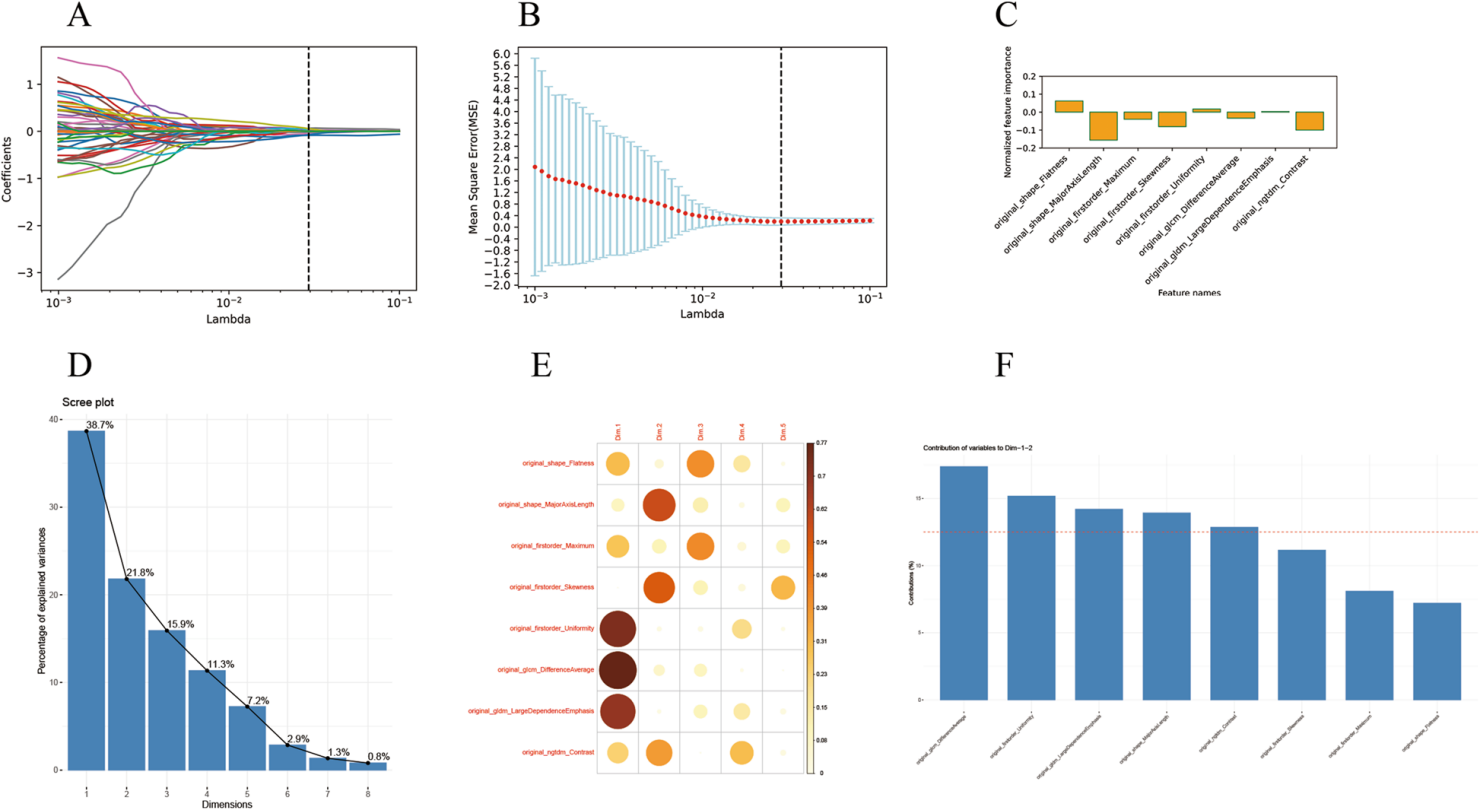
Analyses and selection of radiomics features from T2WI images. **A** LASSO analysis of T2WI. **B** Changes in the automated optimization parameter (λ) when the training samples were fitted with LASSO cross-validation (LASSO-CV). **C** The respective weightage of the initial eight features selected for T2WI. **D** Eight-dimensional PCA analysis showing the percentage of information representing the original data in each dimension. **E** Diagram showing the distributions of the eight initial features within the top five PCA dimensions. **F** Graph showing the percentage contribution by each feature within the top two PCA dimensions. Red line denotes the average contribution percentage. Five features with above average contribution were selected for model generation

**Fig. 4:**
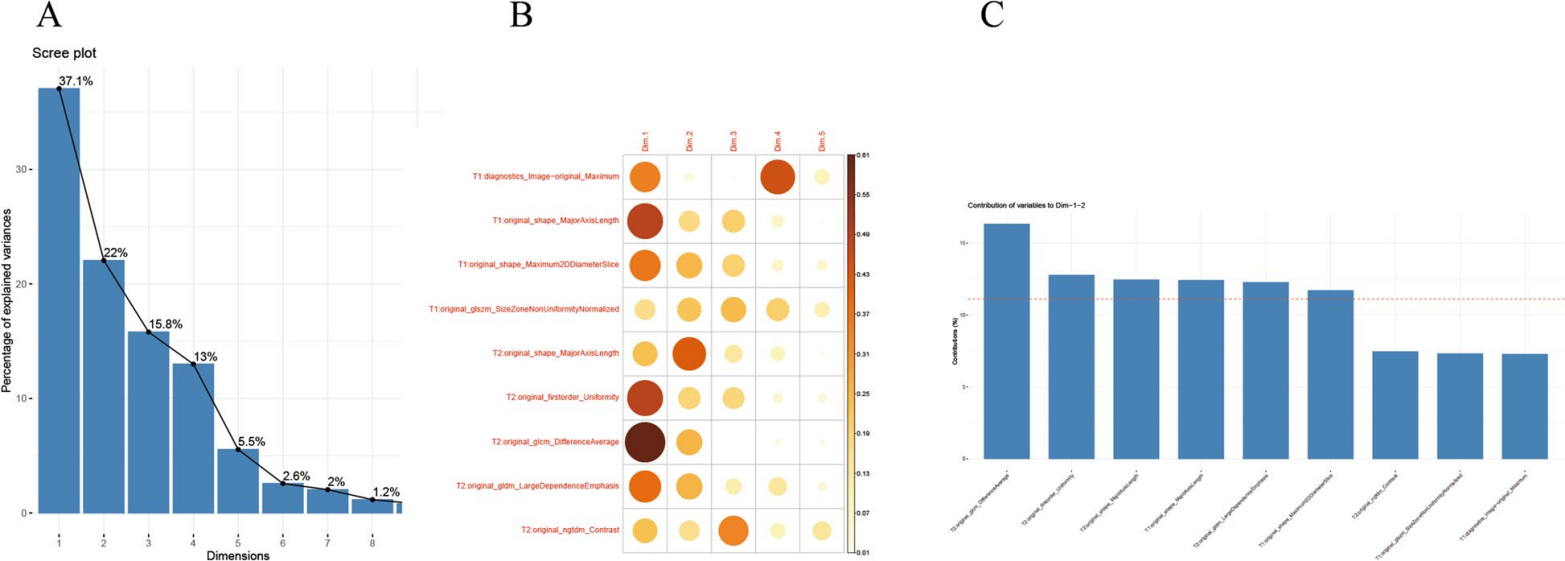
Combined analysis and selection of radiomics features from T1WI and T2WI images. **A** Nine-dimensional PCA analysis showing the percentage of information representing the original data in each dimension. **B** Diagram showing the distributions of the nine initial features within the top five PCA dimensions. **C** Graph showing the percentage contribution by each feature within the top two PCA dimensions. Red line denotes the average contribution percentage. Six features with above average contribution were selected for model generation

To identify statistically significant T1WI imaging features between the two diseases groups, a Student’s t-test was carried out, which identified 12 features in the training image set. To enhance the prediction accuracy and avoid overfitting, we performed the LASSO regression for T1WI images (Fig. 2A). The training samples were fitted by LASSO cross-validation (LASSO-CV) and the automated optimization parameter (λ) is shown in Fig. 2B. Finally, four optimized features (diagnostics_Image-original_Maximum; original_shape_MajorAxisLength; original_shape_Maximum2DDiameterSlice; original_glszm_SizeZoneNonUniformityNormalized) were selected for further modeling and their weightage is shown in Fig. 2C. Similar analyses were performed for the T2WI images. Here, 51 features showed significance between the 2 diseases in the Student’s t-test, which were narrowed to 8 with a LASSO regression analysis (Fig. 3A). Fitting of the training set with LASSO-CV and the automated optimization parameter (λ) is shown in Fig. 3B, while Fig. 3C shows the weightage of the eight selected features (original_shape_Flatness; original_shape_MajorAxisLength; original_firstorder_Maximum; original_firstorder_Skewness; original_firstorder_Uniformity; original_glcm_DifferenceAverage; original_gldm_LargeDependenceEmphasis; original_ngtdm_Contrast). To further limit the number of features that would be used to train the predictive model for T2WI to avoid overfitting caused by redundant radiomics features, we performed a principal component analysis (PCA). An eight-dimensional analysis showed that 94.9% of the original image information can be captured within the first five dimensions (Fig. 3D). Figure 3E shows the distributions of the eight selected features within the top five dimensions, indicating that all features can be captured within the first two dimensions. Indeed, five of the eight features (original_shape_MajorAxisLength; original_firstorder_Uniformity; original_glcm_DifferenceAverage; original_gldm_LargeDependenceEmphasis; original_ngtdm_Contrast) contributed above average within the first two dimensions and are the final selection for model construction (Fig. 3F). Features that are significantly different in both T1WI and T2WI images between the patient groups were also analyzed by PCA. A nine-dimensional analysis showed that 93.4% of the original image information can be captured within the first five dimensions (Fig. 4A). Nine features (four from T1WI; five from T2WI) were captured within the top five dimensions (Fig. 4B), and six features (two from T1WI: original_shape_MajorAxisLength; original_shape_Maximum2DDiameterSlice; four from T2WI: original_shape_MajorAxisLength; original_firstorder_Uniformity; original_glcm_DifferenceAverage; original_gldm_LargeDependenceEmphasis) were finally selected based on their above average contribution to the top two dimensions (Fig. 4C). Table 3 summarizes the final selected features.

**Table 3:** Final features selected for model training

Sequences	Features
T1WI	diagnostics_Image-original_Maximum
	original_shape_MajorAxisLength
	original_shape_Maximum2DDiameterSlice
	original_glszm_SizeZoneNonUniformityNormalized
T2WI	original_shape_MajorAxisLength
	original_firstorder_Uniformity
	original_glcm_DifferenceAverage
	original_gldm_LargeDependenceEmphasis
	original_ngtdm_Contrast
T1WI + T2WI	T1: original_shape_MajorAxisLength
	T1: original_shape_Maximum2DDiameterSlice
	T2: original_shape_MajorAxisLength
	T2: original_firstorder_Uniformity
	T2: original_glcm_DifferenceAverage
	T2: original_gldm_LargeDependenceEmphasis

### Development of a radiomics feature model

**Fig. 5:**
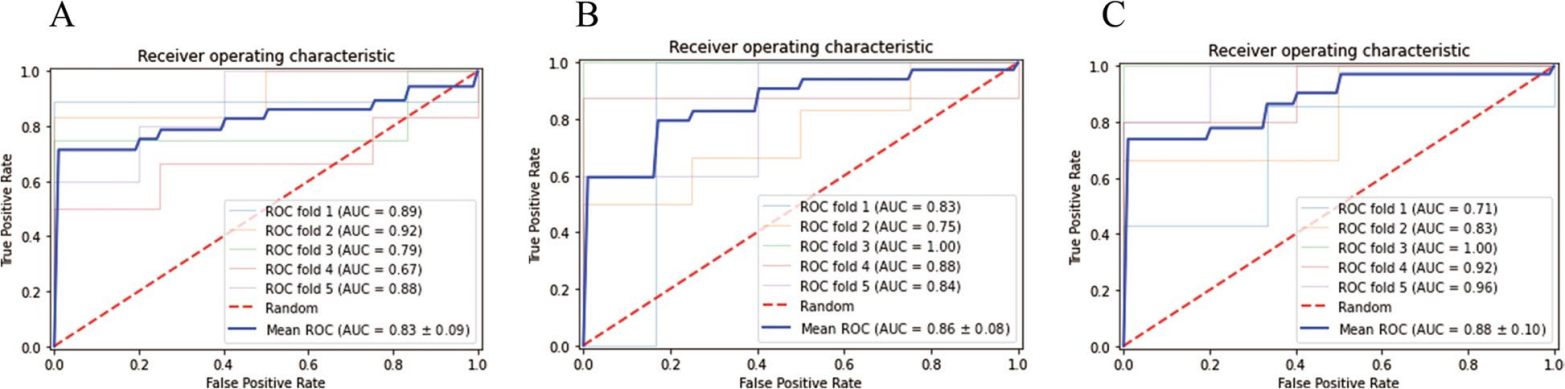
SVM modelling and receiver operating characteristic curves for **A** T1WI, **B** T2WI, and **C** combined T1WI and T2WI features. All curves were generated from the training data set

SVM prediction models were constructed using the features described in Table 3 and the radial basis function (RBF) kernel. Figure 5 shows the receiver operating characteristic (ROC) curves for T1WI (Fig. 5A), T2WI (Fig. 5B) and T1WI combined with T2WI (Fig. 3C) for the training set. The areas under the curve (AUC) are 0.83 ± 0.09, 0.86 ± 0.08 and 0.88 ± 0.010 for T1WI, T2WI, and combined T1WI and T2WI, respectively. For the validation set, the AUC dropped slightly to 0.722 ± 0.037, 0.744 ± 0.027, and 0.774 ± 0.047 for T1WI, T2WI, and combined T1WI and T2WI, respectively (Table 4). However, the trend is the same with the combined modeling of both T1WI and T2WI features showing slightly better performance, suggesting that the combination of T1WI and T2WI features may achieve better results for differential diagnosis of orbital MALT lymphoma and IgG4-ROD in the SVM predicting models.

**Table 4:** Summary of SVM modelling indicating the ability of each model to differentiate between IgG4-ROD and orbital MALT lymphoma

Model	ACU	PRE	REC	F1	AUC
T1WI	0.722 ± 0.037	0.764 ± 0.047	0.813 ± 0.053	0.764 ± 0.036	0.722 ± 0.037
T2WI	0.750 ± 0.028	0.865 ± 0.038	0.767 ± 0.043	0.797 ± 0.027	0.744 ± 0.027
T1WI + T2WI	0.770 ± 0.049	0.811 ± 0.051	0.843 ± 0.053	0.803 ± 0.046	0.774 ± 0.047

*ACU* Accuracy, *PRE *Precision, *REC *Recall, *AUC* Area under the receiver operating characteristic curve

## Discussion

Differential diagnosis between IgG4-ROD and orbital MALT lymphoma have always been a difficult clinical problem. In this study, for patients with orbital eye diseases, contrast-enhanced MRI, which were not included for radiomics analysis, are less commonly used compared to routine T1WI and T2WI. IgG4-ROD and orbital MALT lymphoma have also been shown to have some differential features in T1WI and T2WI. Thus, we focused our attention on T1WI and T2WI, which were selected for this study to determine whether radiomics analyses can extract more information that will assist physicians with the differential diagnosis of IgG4-ROD and orbital MALT lymphoma.

Previously, infraorbital nerve enlargement was observed in IgG4-ROD frequently [22], while ill-defined tumor margins were significantly associated with orbital MALT lymphoma [6]. IgG4-ROD has a tendency to involve both eyes, while unilateral involvement is more common for orbital MALT lymphoma [6, 23, 24], an observation that is consistent with our data. However, the possibility of gender bias between these two orbital diseases is less clear. While our study and that of Yuan et al. [20], and Olsen and Heegaard [23] are aligned in the insignificant effect of gender, several studies have shown otherwise [6, 24]. Thus, gender differences in IgG4-ROD and orbital MALT lymphoma require deeper investigation. The diagnostic performance of combined features of T1WI and T2WI sequences in this study achieved slightly higher AUCs compared to that of the individual sequences, while the value of performance for T1WI appeared to be slightly lower than T2WI. This is consistent with studies published by Chen et al. [25] and Han et al. [26].

The usage of radiomics in differential diagnosis of IgG4-ROD and orbital MALT lymphoma embodies several advantages. First, as there are few published studies that distinguish IgG4-ROD from orbital Malt lymphoma, this research may shed some light on the differential diagnosis of these diseases through radiomics analysis of MR images. Second, quantitative information extracted from MR images instead of qualitative description of these images will reduce human error in diagnosis. Lastly, objective digital features and repeatable experimental results may help young clinical ophthalmologists to correctly diagnosis these two different conditions and select the appropriate treatment.

There are inevitably some limitations in this study. First, the regions of interest were manually drawn, which were time consuming and may be prone to error. Thus, future studies should focus on performing automated segmentation of the lesions. Second, the limited sample size from a single center is likely to create biases within this study. Although we have restricted the number of optimal features used to construct the predictive models to avoid overfitting, the study can benefit from larger sample size and independent validation data sets. Extensive optimization and validation, involving multi-center studies, will improve the generalizability of our research.

## Conclusions

To improve the differential diagnosis of IgG4-ROD and orbital MALT lymphomas, we have extracted several important radiomics features from T1WI and T2WI MR images. The radiomics models that were generated from these features showed promising predictive values and may provide useful guidance for clinical decision-making. More detailed radiomics features and advanced techniques should be explored to further improve the differential diagnosis of IgG4-ROD and orbital MALT lymphomas.

## Data Availability

The raw data supporting the conclusions of this article will be made available by the authors without undue reservation. All data can be requested from author Yuchao Shao.

## Abbreviations

MALT: mucosa-associated lymphoid tissue
IgG4-ROD: IgG4-related orbital disease
MRI: magnetic resonance imaging
T1WI: T1-weighted images
T2WI: T2-weighted images
LASSO: the least absolute shrinkage and selection operator
PCA: principal component analysis
SVM: support vector machine
AUC: area under the curve
ROC: receiver operating characteristic
TAO: thyroid-associated ophthalmopathy
RBF: radial basis function
CV: cross validation
ICC: interclass correlation coefficient
ACU: accuracy
PRE: precision
REC: recall.
